# Octocoral Species Assembly and Coexistence in Caribbean Coral Reefs

**DOI:** 10.1371/journal.pone.0129609

**Published:** 2015-07-15

**Authors:** Johanna Velásquez, Juan A. Sánchez

**Affiliations:** Departamento de Ciencias Biológicas-Facultad de Ciencias, Laboratorio de Biología Molecular Marina (BIOMMAR), Universidad de los Andes, Carrera 1E No 18A–10, Bogotá, 111711, Colombia; Biodiversity Research Center, Academia Sinica, TAIWAN

## Abstract

**Background:**

What are the determinant factors of community assemblies in the most diverse ecosystem in the ocean? Coral reefs can be divided in continental (i.e., reefs that develop on the continental shelf, including siliciclastic reefs) and oceanic (i.e., far off the continental shelf, usually on volcanic substratum); whether or not these habitat differences impose community-wide ecological divergence or species exclusion/coexistence with evolutionary consequences, is unknown.

**Methods:**

Studying Caribbean octocorals as model system, we determined the phylogenetic community structure in a coral reef community, making emphasis on species coexistence evidenced on trait evolution and environmental feedbacks. Forty-nine species represented in five families constituted the species pool from which a phylogenetic tree was reconstructed using mtDNA. We included data from 11 localities in the Western Caribbean (Colombia) including most reef types. To test diversity-environment and phenotype-environment relationships, phylogenetic community structure and trait evolution we carried out comparative analyses implementing ecological and evolutionary approaches.

**Results:**

Phylogenetic inferences suggest clustering of oceanic reefs (e.g., atolls) contrasting with phylogenetic overdispersion of continental reefs (e.g., reefs banks). Additionally, atolls and barrier reefs had the highest species diversity (Shannon index) whereas phylogenetic diversity was higher in reef banks. The discriminant component analysis supported this differentiation between oceanic and continental reefs, where continental octocoral species tend to have greater calyx apertures, thicker branches, prominent calyces and azooxanthellate species. This analysis also indicated a clear separation between the slope and the remaining habitats, caused by the presence or absence of *Symbiodinium*. K statistic analysis showed that this trait is conserved as well as the branch shape.

**Discussion:**

There was strong octocoral community structure with opposite diversity and composition patterns between oceanic and continental reefs. Even habitats with similar depths and overall environmental conditions did not share similar communities between oceanic and continental reefs. This indicates a strong regional influence over the local communities, probably due to water transparency differences between major reef types, i.e., oceanic vs. continental shelf-neritic. This was supported by contrasting patterns found in morphology, composition and evolutionary history of the species between atolls and reef banks.

## Introduction

Since the time of Darwin, it is known that coral reefs can differ regionally due to their history of geological formation (e.g., atolls, barrier reefs, fringe reefs, banks), which in turn can lead to the formation of a diversity of local habitats (e.g., lagoon, fore-reef, slope, plateau) [1]. In both cases, environmental variables differ over depth gradients geographic locations and geological histories. At a regional level, coral reefs can be divided in continental, i.e., reefs that develop on the continental shelf, and oceanic, i.e., far off the continental shelf, usually on volcanic substrata. Community studies in the Western Caribbean report a compositional separation between these two reef types, mainly due to the impact of terrestrial run-off sources over continental coastal reefs, and a greater rate of disturbances in oceanic reefs [2]. Although terrestrial run-off can provoke well known adverse effects on corals and coral reefs (see review in [3]), some coral reefs successfully develop along neritic coastal siliciclastic-environments [4]. Whether or not these diverse conditions impose community-wide ecological divergence or species exclusion/coexistence with evolutionary consequences, is unknown.

Species’ coexistence within a community is the result of the interactions among species as well as their phenotype-environment relationship [5]. Historically, phenotypic resemblance among species were thought as traits that evolved from a common ancestor, that in other words, reflects their evolutionary history [6, 7]. This results in ecological similarities that determine a preference of habitat, which favors the coexistence among similar species, or, on the contrary, intensifies competition, which excludes species from particular assemblages [6]. Rooted in these assumptions, both hypothetic cases stand as the most important principles for explaining the assembly, diversity and composition of communities [8, 9], where environmental heterogeneity and evolutionary tradeoffs determine species coexistence [9]. Therefore, the community assemblage we see today is the product mainly of dispersion and competition abilities.

At a local scale, competition is undoubtedly the main toll for species exclusion or coexistence in a community [10]. There are exemplary cases of niche partitioning to avoid competition in marine communities. Barnacle communities in temperate waters (4–6 species per assemblage), for instance, display character displacement along the ramus length, a functional trait related to feeding preferences [11]. However, on species-rich tropical communities, such as coral reefs with at least an order of magnitude more coexisting species than temperate rocky shore ecosystems, it is improbable to find that level of niche partitioning. Moreover, it has been suggested that the sessile states of reef coral communities, similar to tropical trees, have overall weak effective interactions [12]. The local community, in species-rich ecosystems, has been suggested as an epiphenomenon with little meaningful information on species coexistence [13]. Without denying that there is always antagonic interactions and competition within species assemblages that affect species exclusion/coexistence ([10], see [14] for a review in plants), our study focuses on determining the phylogenetic community structure in a octocoral reef community emphasizing insight on species coexistence evidenced with trait evolution and environmental feedbacks.

At a regional scale, ecological communities are thought to influenced by biogeographic and historic processes [15, 16]. Phylogenetic overdispersion at narrow spatial and taxonomical scales is predominant and it is assumed that closely-related species at a smaller scale are prone to competition as opposed to less related and geographically isolated taxa [17]. Then, the most appropriate approach is to ask where do species have similar ecological requirements and dispersion possibilities between communities [18], which can become evident in the species’ functional traits [19], through the examination of ecological and geographical gradients. With the use of phylogenies, morphological traits, and environmental preferences we can understand which patterns and processes are responsible for species distributions [7]. Under the niche theory, if the coexistence of phenotypically similar species (phenotypic clustering) occurs because of a strong environmental filter, it can lead to a phylogenetic clustering if the traits are conserved, or to a phylogenetic overdispersion if trait convergence corresponds to less related species [7]. A strong habitat filter, if evident by the marginal environmental conditions in the metacommunity, can impose relative fitness differences leading to small niche differences, which prevent competitive exclusion and promote species coexistence [10]. Alternatively, if phenotypic similarity entails an increase in the pressure of interspecific competition, this will result in competitive exclusion. Consequently, this would lead to communities formed by phenotypically more dissimilar species. Phylogenetically, they can be clustered or overdispersed, depending also on trait evolution [7]. A functional equivalency between species has also been suggested, where the relative abundances of species depends mainly on dispersion capabilities [20]. In this neutral theory, despite that communities are conceived as the sum of many biotic and abiotic interactions, it has been possible to explain the structures of different community types, mainly in plants [21]. Patterns and processes that lead to the assembly of communities in coral reefs are still controversial under the niche and neutral theories [12, 22, 23].

Typical Caribbean reef dwellers, octocorals (Cnidaria: Octocorallia) in this region (commonly known as gorgonian corals) belong mostly to the families Gorgoniidae and Plexauridae, and form colonies that are normally erect, branching and with a defined axis or medullar zone, which, among other morphological traits as the sclerites, have resulted in the basis for species identification. Being sessile organisms in their adult phase, dispersal will depend on the larval and gametic phases; therefore both their survival and ability to settle will be essential in the species assembly. For example, it has been found that larvae of broadcast-spawning species have a tendency to disperse farther away but have lesser survival rates than brooder species [24]. However, these colonization and settlement rates not only depend on the larva’s ability to disperse, but also on the physical and biological barriers [25]. A phenotype-environment mismatch can prevent the recruits' settlement to nearby areas of species with high capacity of dispersion [26]. Various studies have shown that these organisms, at a local level, have a community structure that follows zoning patterns, guided mainly by depth, light penetration, water movement and sediment transportation gradients [27]. It has been found that many species have a limited range of distribution, where normally the abundance [28, 29] tends to be greater in some habitats like the fore-reef terrace, indicating that the conditions at certain habitats can be ideal for octocoral growth and recruitment [27, 28, 30–32].

This arguable specialization of habitat allows the coexistence in sympatry of many species in a community. Most abundant shallow-water octocorals have symbiosis with dinoflagellate microalgae, commonly known as zooxanthellae (*Symbiodinium*) [30, 32–34]. However, this mutualism renders the corals vulnerable to bleaching [35]. This is also a driving force structuring coral reef communities because species have to establish in places within certain ranges of environmental conditions [31, 33]. For azooxanthellate shallow-water octocorals (heterotrophic), it has been noticed a greater abundance in places with low light intensities [28, 30], which is associated with depth, high productivity and food availability [36]. Finally, it is important to highlight that, although morphological traits have been important in the identification of soft corals, their phenotypes are subject to changes caused by environmental variation [37], which has have related to ecological speciation [38, 39]. The relevance of this study is given that it aims to establish the key factors in the assembly of coral reef organisms, using octocorals as model system. Here we determine the role of evolutionary history, environment and the evolution of traits determining the coexistence and distribution of species.

## Material and Methods

### Sample data

**Fig 1:**
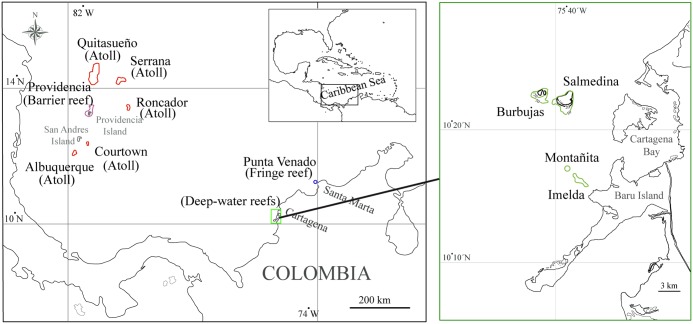
Geographic distribution of sampled coral reefs in the Western Caribbean (Colombia). Each color represents a different reef type: four atolls, one barrier reef, four reef banks and one fringe reef (map contours were tablet-digitized from the nautical charts COL 005 and 261, CIOH, República de Colombia).

We evaluated 11 octocoral communities (Phyllum: Cnidaria, Subclass: Octocorallia) in the Colombian Caribbean Sea (Fig 1), where, with the exception of five sites, the abundance data had been previously obtained. These communities are distributed at different reef complexes including: one fringe reef in Santa Marta (Punta Venado), four continental shelf reef banks in Cartagena (Montañita, Imelda, Salmedina and Burbujas), one barrier reef in Providencia Island [30] and five atolls; Serrana, Quitasueño and Roncador [27], and Albuquerque and Courtown (which were analyzed together) [28].

The five atolls in the San Andres and Providencia Archipelago, together with the barrier in Providencia Island, are oceanic reefs. Both types of reefs originated from volcanic islands, most likely in the early Cenozoic [40]. Barrier reefs extend several kilometers away from the coast in a parallel manner, whereas atolls tend to grow in a circular shape, as isolated emerging seamounts [41]. Their geomorphologic setup is mainly conformed by a lagoon with 8 to 15 m deep harboring parch reefs, and a bottom of fine sand and seaweed, where the sediments from peripheral reefs are gradually deposited. They also have a reef crest, which usually absorbs the energy of breaking waves [42] and a fore-reef, which reaches about 30 m deep, and is characterized by having a gradual inclination, spur and groove systems and hard substrate irregularity [2]. Continental reefs develop on the continental shelf, e.g, reef banks, and some develop right next to the rocky shoreline, e.g., fringe-reefs [41]. Reef banks comprise ancient diapiric domes, which were progressively colonized by corals [43], consisting of two types of habitats: the plateau and the slope.

Since different methods of species counting were used in the original data sources, colony abundance was standardized to obtain a 10 m^2^ species density for each habitat and reef. For the communities sampled in September and December, 2010 (in the reef banks), a transect of 50 meters was established in each habitat, and the number of species in a square meter was counted with a 50 cm quadrant (two quadrants for each side of the transect), with 5 m intervals [31]. Once we had the matrix of abundance for each community, a similarity analysis with the Bray-Curtis method was made, as well as a Simper analysis for calculating the contribution percentage of groups formed in the dendrogram. With the exception of the diversity indexes among reefs, the mean abundances by reef and habitat were used for the remaining analysis (see S1 Table for raw data).

### Phylogenetic reconstruction

All available sequences were retrieved from GenBank including the mitochondrial genes mtMutS and ND2. It was necessary to obtain new DNA extractions, amplifications, purifications and sequences for 14 out of the 49 species included in the study. For this purpose, we took tissue from samples deposited in the Museum of Natural History of Universidad de los Andes (ANDES). Additionally we collected fresh samples in field trips to the Cartagena area. DNA extraction followed the CTAB protocol [44], and PCR and sequencing were according to [45]. New DNA sequences, until now unpublished, were possible thanks to Genetic resources Contract 007, resolution 634, 14 March 2007, MADS and specimen collections thanks to Biodiversity Research permits DTC-CR-T-036-03-09 and DTC-LCR-002-2004 (MADS-Ministry of Environment and Sustainable Development, Colombia).

For phylogenetic trees reconstruction, sequences were edited and concatenated in Geneious 5.3. Using this same software, they were aligned using the MUSCLE algorithm. PAUP* 4.0 [46] was used for the construction of a maximum parsimony tree, by means of a heuristic search and bootstrapping support from 1000 replicates. Nucleotide evolution models for the maximum likelihood-ML and Bayesian inference-BI trees were selected using MrModelest 2.3. The selected models were GTR+I+G for ML and HKY for ND2 and HKY+G for mtMutS in BI. The maximum likelihood tree was obtained using RAxML using two partitions (this way the algorithm recognizes when a different gene began, thus accounting for missing sequences) and a bootstrap resampling with of 1000 repetitions. The Bayesian inference tree was run with partitions for each gene, ten million generations for MCMC and a 25% burn-in using MrBayes. Forty-nine species, represented in five families, conformed the species pool (see S1 Fig).

### Environment vs. local and regional diversity

The Shannon´s diversity indexes for the local diversity and the Sorensen index for the regional diversity were calculated with the the Picante and Vegan R packages, using the abundance by reef matrixes and the average by habitat (for the same habitat of different reefs we calculate the average of the abundance). The phylogenetic diversity (PD) index [47] and the phylobetadiversity [48] were calculated using the inferred phylogenetic tree. Species contribution percentages were established for each group formed by the Sorensen similarity analysis using the Primer 6 software. On a local level, we tried to establish the relationship between species diversity and phylogenetic diversity, through a Pearson correlation. For the phylobetadiversity analysis, the phylogenetic community dissimilarity (PCD) metrics were calculated, which derivate from two components: compositional (PCDc), which is analog to Sorensen’s but correcting the effect that causes the community’s size, and phylogenetic (PCDp), which measures the phylogenetic relation of species that are not shared between communities. To estimate the correlation between regional diversity indexes and the environmental variables that can be implicated in structuring communities, we obtained data of the reef’s areas, the distance between reefs, the distance between the reef and the continent, the mean depth of each habitat and the mean area of each habitat. Distances were calculated with Google Earth, depths were registered directly in the field and areas were calculated using ImageJ (Java version of NIH image) from scaled maps in the literature [27, 28, 30, 32]. A Mantel test was performed to estimate the Pearson correlation between each metric and each variable. Positive relationships between environmental variables and either the PCDc or PCDp were examined to test if similar environments share similar species and if non-shared species tend to belong to the same clade [48]

### Phenotype-environment relationship

Two different analyses were carried out to testing whether there was a phenotypic clustering or overdispersion. For both cases, an eight-trait matrix was built (see S2 Table for raw data). To achieve this, four discrete characters and four continuous characters, were evaluated for each species. For the discrete characters, the traits were extracted from the literature [49, 50], which included: colony shape (encrusting, flagelliform, pinnate, candelabrum-like, bushy, fan-like or feather-like), branch shape (cylindrical, flattened or no branching), branching plane (single, multiple or no branching) and the presence/absence of zooxanthellae. For the continuous data, all samples deposited in the Museo de Historia Natural of Universidad de los Andes (ANDES) that were collected at the research sites were used. Four photographs were taken of each colony (the number of colonies per species was depending on the available quantity in the museum), covering 4 cm of colony for each side of a terminal branch. Using ImageJ, 10 measurements per character were taken and finally an average was calculated. The evaluated characters for that case were: branch thickness, calyx length, intercalyce distance and calyx aperture.

After collecting the data, two analyses were performed. First, a discriminant components analysis was made in the program IBM SPSS Statistics 19, discriminating the data by habitat and reef. Here we use those species that were notably more abundant in a certain type of habitat or reef (abundance was at least the double of the next higher abundance). Second, a SESmetric was calculated with 1000 randomizations using Phylocom 4.0.1.b [51] and all abundance matrix (not only the most abundant species), where values above zero indicate phenotypical clustering and values below zero overdispersion. A two-tailed t-test with 95% confidence intervals was conducted, for inquiring if the results were considerably different from zero.

### Phylogenetic community structure

In order to test for phylogenetic clustering or overdispersion, the net relatedness index (NRI) and the nearest taxon index (NTI) were estimated, which represent the difference between the mean phylogenetic distance in observed and null communities [7]. This analysis was run 1000 times in Phylocom 4.0.1.b (Webb, Ackerly et al. 2008), with a null model that maintained the frequency of species for each community. Both indexes were calculated for the type of habitat and the type of reef, using the ‘Comstruct’ command (with the abundances and the phylogeny described above). Positive indexes support phylogenetic clustering hypothesis and negative support indexes phylogenetic overdispersion [7]. To estimate if they were considerably different from zero, a two-tailed t-test with 95% confidence intervals was conducted.

### Traits evolution

To determine if the eight studied traits were conserved or labile, according to what was expected under a Brownian motion model of evolution, the K statistic was calculated [52] using the Picante package for R. With values >1 traits were considered conserved, < 1 labile, and with values near 1 under Brownian motion [52].

## Results

### Environment vs. local and regional diversity

At a local scale, a positive correlation was found between Shannon’s index and phylogenetic diversity depending on habitat type (*P* = 0.0184, R^2^ = 0.31, B = 0.09), where the highest values for both indexes were present in the fore-reef and plateau habitats (above 2 for Shannon’s index and above 0.55 for phylogenetic diversity). For reef type, there was no correlation (*P* = 0.600, R^2^ = 0.058, B = -0.041). Highest values for the Shannon index were found in atolls and barrier reef (values above 2.6), whereas phylogenetic diversity was higher in reef banks (values above 0.58).

**Fig 2:**
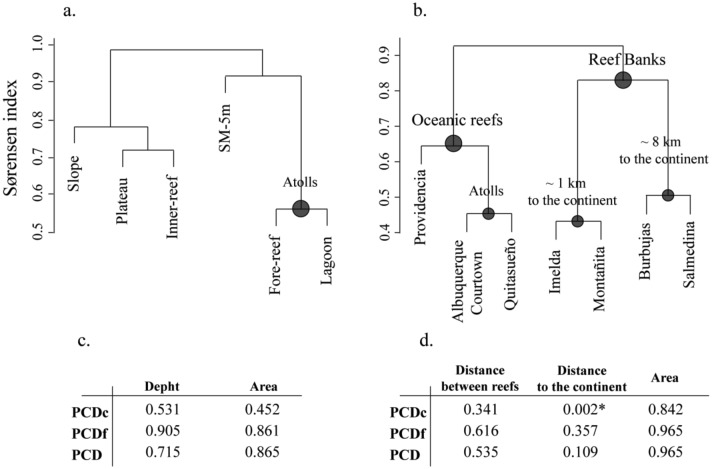
Sorensen beta diversity between habitat type (a) and reef type (b) for Caribbean octocoral communities. Circles represent groups that can be explained by distribution patterns. *P*–value of Pearson’s correlation with Mantel test of phylogenetic community dissimilarity and environmental condition, between habitat type (c) and reef type (d). *Significant correlation.

As for regional diversity, the compositional structure of communities with respect to habitat and reef type did not differ between the Sorensen’s index and the PCDc (Fig 2, 2a and 2b; Sorensen index not shown). For habitat type there was no association between the communities’ composition (for both Sorensen index and PCDc) and distribution patterns (Fig 2a) or environmental variables (Fig 2c). On the other hand, the grouping according to reef type showed a clear representation of oceanic and continental reefs (Fig 2b). Furthermore, reef banks were arranged according to their proximity among them and to the coast (Figs 1 and 2b). Moreover, these results where statistically supported by the PCDc and the distance to the coast matrix correlation (Fig 2d). Both for habitat type and reef type, there was an association between the evaluated variables and the PCDp, which means that species not shared among communities were from different clades in a random way (Fig 2c and 2d).

Simper analysis determined that the members with a higher contribution for the formation of the group between fore-reef, lagoon and SM-5m were three *Antillogorgia* species (*A*. *acerosa*, *A*. *americana* and *A*. *bipinnata*), with a sum of 73% of contribution. For the group that made up the slope, inner-reef and plateau, the species with a higher contribution was *Eunicea flexuosa* with a 61%. Depending on reef type, the oceanic reefs were characterized mainly by the presence of *A*. *bipinnata* and *Briareum asbestinum* (74%), whereas the continental ones were characterized by the contribution of *E*. *flexuosa* and *E*. *clavigera* (56%).

### Phenotype-environment interaction

**Fig 3:**
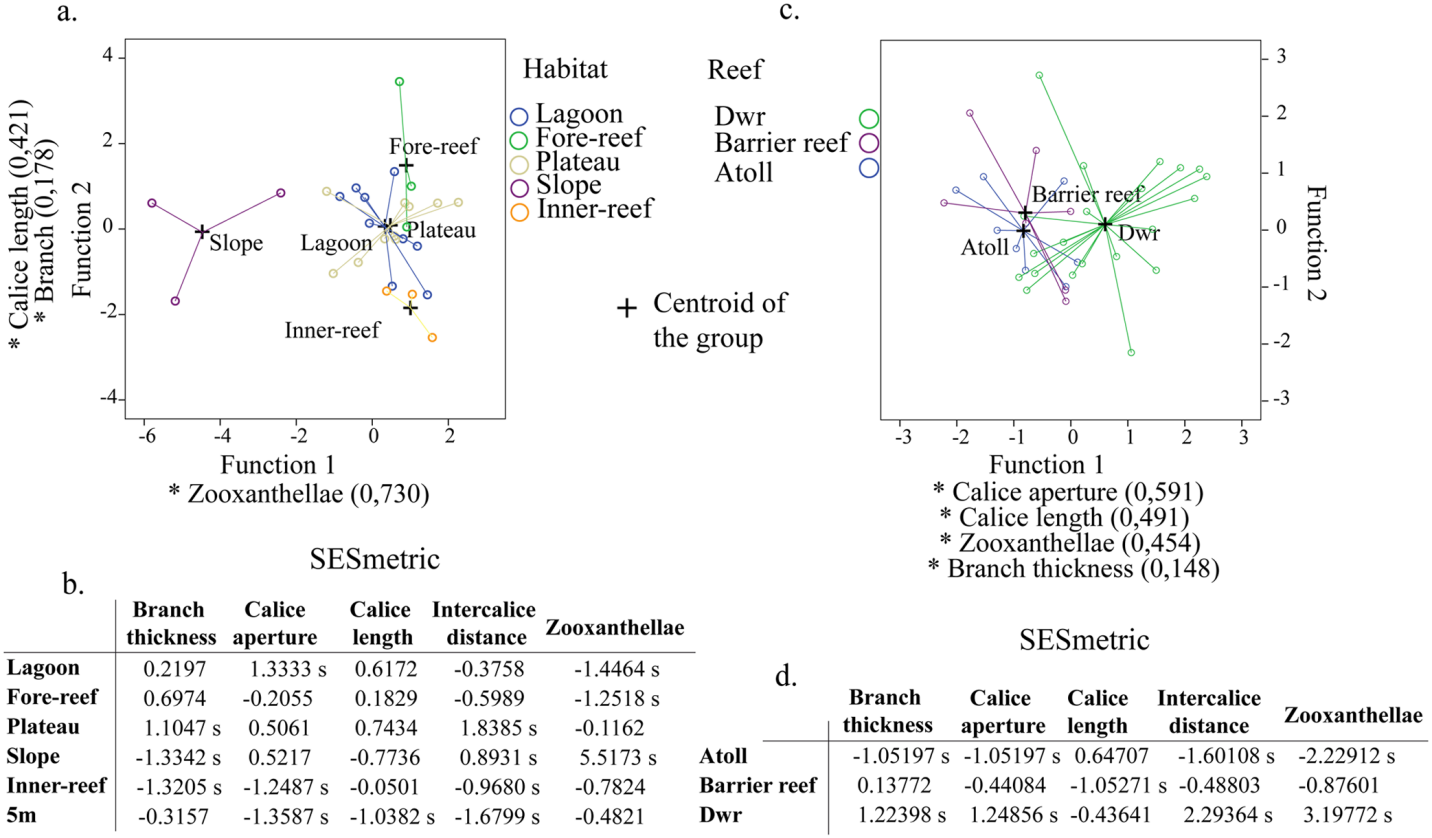
Discriminant component analysis, for the most abundant Caribbean octocoral species in every sample, to determine morphologic similarities according to the habitat type and reef type. For the SM_5 m habitat neither species was more abundant with regard to the rest of the habitats. SESmetric for the four continuous and one binary traits (the analysis does not allow to run multistate traits) according to habitat type (c) and reef type (d). * = Greater absolute correlation between each variable and any discriminant function; s = Data significantly different from 0 with two-tailed t-test and 95% confidence interval.

The discriminant component analysis for the eight traits and filtering by habitat type (Fig 3a) showed a clear separation between the slope and the remaining habitats, explained by the presence/absence of zooxanthellae. Furthermore, there seems to be a separation between the fore-reef and the inner-reef, because species in the first habitat tend to have flattened branches and short calyces, whereas in the inner-reef they grow cylindrically and with longer calyces (Fig 3a). For reef type (Fig 3b), there was again a separation between oceanic and continental reefs, where continental species (reef banks) tend to have greater calyx apertures, thicker branches, prominent calyces and azooxanthellate species.

Branch thickness in the species found in the plateau habitat was more uniform than expected by chance, but it was very different in the species from the slope and inner-reef (Fig 3c). As for calyx aperture, lagoonal species were more similar to each other, whereas SM_5 m habitat and inner-reefs species were more dissimilar. The calyx length only resulted phenotypically overdispersed for the SM_5 m habitat. For intercalyce distance, phenotypical clustering was found in the plateau and phenotypical overdispersion was found in the SM_5 m habitat. Finally, with respect to the presence of zooxanthellae, all the habitats were uniform, with the exception of the plateau. On the other hand, for reef type, results were more consistent for all variables (Fig 3d). In general, it was found that in all cases (with the exception of calyx length) species in atolls were less similar among each other, whereas in reef banks it was the opposite.

### Phylogenetic community structure

**Fig 4:**
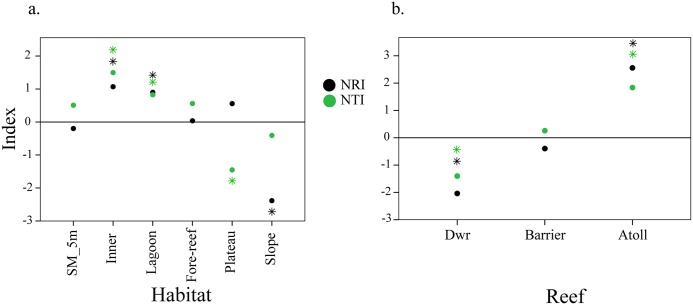
Results from the nearest-taxon (NTI) and net-relatedness (NRI) indexes. NTI for habitat type (a) and reef type (b) for Caribbean shallow-water octocoral communities. * Values significantly different from 0 under a two-tailed t-test and interval 95% confidence interval.

Results from both NTI and NRI indexes suggest a phylogenetic clustering in the shallowest reefs (with the exception of SM-5 m habitat). On the contrary, a phylogenetic overdispersion was found in reef banks habitats, which was statistically significant for NTI in the plateau and NRI in the slope (Fig 4a). When evaluating reef type atolls and reef banks presented opposite patterns, with phylogenetic clustering observed in the atolls and overdispersion in the reef banks (Fig 4b). The fore-reef and the SM-5 m habitats together with the barrier reef did not exhibit phylogenetic structure (Fig 4).

### Traits evolution

K statistic analysis (see S3 Table) showed that presence/absence of zooxanthellae as well as the branch shape (cylindrical, flattened or non-ramified branches) were conserved traits (K statistic > 1). Other traits (branch plane, colony shape, branch thickness, intercalyce distance, calyx length and calyx aperture) had a K statistic < 1, showing that the traits were labile.

## Discussion

All the evidence gathered (phylogenetic, abundance and trait) supported a strong community structure with opposite ecological-evolutionary patterns between oceanic and continental reefs. Even habitats with similar depths and overall conditions (e.g., temperature) did not share similar communities between oceanic and continental reefs. This indicates a strong regional influence over the communities, probably due to water transparency differences between major reef types, i.e., oceanic vs. continental shelf-neritic [2]. This was supported by contrasting patterns found in morphology, composition and evolutionary history of the species between atolls and reef banks. Essentially, ecology-evolution feedbacks played a different role in the formation of the communities in each reef type.

### Phylogenetic relationships and traits evolution

As in previous octocoral studies [45], most of the traits turned out to be labile and convergent, which explains the little correspondence between morphology and phylogeny. It has been proposed that environmental gradients influence coral species morphology, based on observations that incipient species usually live in different habitats [38, 39]. These differentiation could lead to ecological speciation [38]. Octocorals are modular organisms where integration/disintegration or coupling/decoupling within the polyp and branch characters allow species to acclimatize and ultimate to adapt to different environments [37, 53], which could explain the high levels of traits convergence.

Two traits were conserved according to the K statistic analysis: the presence/absence of zooxanthellae and branch shape (flattened, cylindrical or no branching growth). This was also evident in the phylogenetic tree that shows a clade grouping species without zooxanthellae belonging to multiple families. Both traits could be considered important in a phylogenetic clustering context because of the possibility of involving phylogenetic niche conservatism, where the species were adapted to their habitat through their evolutionary history [7, 54].

### Patterns of octocoral species coexistence at local scale

Octocoral assemblages from similar (depth, temperature, current) habitats (e.g., fore reef and plateau; oceanic and continental respectively) had similar species diversity and phylogenetic diversity, but exhibited different community composition, which is suggestive of a strong regional effect on the community structure. However, the fore-reef terrace (with the exception of zooxanthellate-symbiosis presence) turned out to have morphologically and phylogenetically randomly distributed species. Apparently, this habitat has the ideal conditions for many species, which can be evidenced in the comparatively high diversity indexes of this habitat with respect to others [28]. For sessile marine organisms, especially octocorals with most of their biomass above the substrate, it has been determined that competition is unlikely to be deterministic, and so the coexistence of species is promoted [55]. Whereas in the plateau, a habitat conformed by siliciclastic reefs, species are more similar in terms of branch thickness and distance between calyces (thicker with separate calyces), which can be associated with a photosynthetic optimization due to poor water transparency and/or for maximizing filter-feeding in more nutrient-rich conditions. Similar evidence has also been observed in scleractinian corals, where their width to height ratio [56] and a rapid growth to avoid sediment suffocation [38]. These observations are congruent with the ‘habitat filtering’ scenario, where small relative fitness differences promote coexistence.

Habitats with extreme conditions for octocorals, such as the lagoon, led to the coexistence of some specialized but related species. We found both phylogenetic and phenotypic clustering regarding the calyx aperture as well as the presence of zooxanthellae, where the first trait was labile and the second conserved. The effect of habitat filtering was also noticeable, where species with similar calyx apertures are being selected. It has been suggested that the characters of the polyp (calyce aperture) tend to evolve independently to those of the branches (intercalyce distance and branch thickness) [57]. This also supports Porter’s model [58], where it is suggested that corals optimize the light-capture microclimate with small polyps and thin branches. Interestingly, the dominating genus (*Antillogorgia*) presents all the described characteristics. Octocoral species with smaller calyces were identified by the Simper analysis in the lagoon. This habitat is unsuitable for many species but favorable for others (such as *Antillogorgia* species) [59]. We can consider this case an example of ‘phylogenetic niche conservatism’ [54], where as more space for settlement is available, a stabilizing selection occurs, leading to the diversification of related and similar species [60]. Atoll´s lagoon exhibit and increase in oxygen-depletion stress with depth [61] and a documented record of mass mortalities in corals and other benthic organisms [62, 63].

Favorable conditions can induce related species to adopt different morphologies to avoiding competition. Habitats from fringing reefs, such as the inner reef, the closest to the continent, turned out to be phenotypically overdispersed for branch thickness and calyx aperture but phenotypically conserved for cylindrical branch shape (this last trait condition can maximize the light-capture microclimate in turbid waters [58, 64, 65]). The phenotypical overdispersion may be explained by the use of different strategies to withstand this habitat and the difficulty to colonize other habitats, where for example, some species do better being thicker and optimizing photosynthetic rate [64], whereas others can be thinner to overcome sediment suffocation and growing faster [38]. In addition, and because the high levels of sedimentation, some species could have large polyps to optimize heterotrophic feeding, while others may prefer to reduce them to optimize light capture [58]. Therefore, in this case there is a combination of adjustments for both polyp and branch traits, in order to coexist, which may be considered a ‘trade off’ mechanism [9].

The slope, the deepest habitat studied, has the larger SESmetric value regarding the presence/absence of zooxanthellae, in which the species without symbiotic associations clearly dominate. Some of the species with zooxanthellae in this habitat implement different strategies to endure the challenges that deeper depths and more severe slopes represent [38], such as settling under foliose coral substrates [32, 66]. This can be a case of ‘niche evolution’ because the species colonize and persist in a new habitat [67]. But then, why symbiotic species colonize these places, being a habitat with stressful conditions? It is possible, that as suggested for hard corals [68], these species encounter less competition and more available space.

### Patterns of octocoral species coexistence at regional scale

Overall, the patterns found for all analyses at a regional scale had a better congruence, so conclusions are more revealing. The regional influence on the octocoral community structure was very strong for two reasons. First, the composition by reef type was clearly differentiated depending on whether it was an oceanic or continental reef. However, a spatially continuous neutral model cannot be assumed, because a significant correlation between PCDc and the distance between reefs was not established [69]. This assumption would indicate that, due to dispersion, closer reefs are more similar in composition [69].

Second, a significant correlation was found between the distance of each reef to the continent and the PCDc. Oceanic reefs have higher species diversity whereas reef banks have more phylogenetic diversity. The phylogenetic component to determine the regional phylogenetic dissimilarity (PCDp) and environmental variables had no significant correlations, which suggests that species not shared among communities do not tend to be from the same clade, nor from statistically different clades, suggesting a random pattern [48]. Reef banks have a very high phylogenetic diversity, where coexistence is promoted for distantly related but morphologically similar species. Atolls were very different presenting phylogenetic clustering and phenotypic overdispersion, showing that species could be avoiding competition. Barrier reefs seem to have optimum conditions that allow species to exploit resources equitably in their multiple habitats. It has intermediate environmental conditions between atolls and reef banks, allowing for random colonization of many species.

## Conclusions

There was a clear local and regional influence over the octocoral community structure in Caribbean reefs, where, as with other marine invertebrates, competition does not seem to have an important role in structuring communities [55, 70]. It seems that turbidity and water motion are highly linked to the assembly of species. In many cases octocoral species exhibit morphological plasticity to overcome these conditions by means of adjusting polyp and branch characters, which given the contrasting conditions provided by the different reef and habitat types from Caribbean reefs can promote ecological speciation [71].

The changes that marine ecosystems are facing today are unprecedented; despite that species richness has remained constant in the last decades, the composition of species assemblages is changing worldwide [72]. This result has raised new concerns on marine conservation given that this species turnover corresponds to less favorable ecosystem states in terms of productivity and services. However, it is worrisome how little we understand species coexistence in marine ecosystems such as coral reefs. This study highlights the importance of comparative studies to understand patterns of species distributions and community assembly in marine ecosystems.

## Supporting Information

S1 Fig Phylogenetic tree of shallow-water octocorals in the Caribbean based on partial ND2 and mtMutS sequences. The represented is the unrooted Bayesian Inference tree with its respective branch supports (estimated posterior probabilities). The bayesian inference, maximum credibility tree was the one with the highest branch support and best resolution in terminal branches although phylogenetic relationships among the three methods did not differ. Sequences that end with J were obtained in this study. (*Pseudopterogorgia* = *Antillogorgia*).
(DOCX)

S1 Table Octocoral community abundances. Data standardized to obtain a 10 m^2^ species density for each habitat and reef (see methods for details). A. Abundances by reef. B. Abundances by habitat.
(DOCX)

S2 Table Functional characters data. Colony shape, Branch type, Branching plane and Zooxanthellae presence are discrete qualitative characters. Branch thickness (cm), Calice length (cm), Intercalice distance (cm) and Calice aperture (cm) are quantitative mean values.
(DOCX)

S3 Table Eight evaluated traits with their respective categories, K statistic and interpretation of their evolution. (DOCX)
