# Supplementary material for: Octocoral Species Assembly and Coexistence in Caribbean Coral Reefs
Source: PLoS One. 2015 Jul 15;10(7):e0129609. doi: 10.1371/journal.pone.0129609 (PMC4503594; doi:10.1371/journal.pone.0129609)
Supplement: S1 Fig — The represented is the unrooted Bayesian Inference tree with its respective branch supports (estimated posterior probabilities). The bayesian inference, maximum credibility tree was the one with the highest branch support and best resolution in terminal branches although phylogenetic relationships among the three methods did not differ. Sequences that end with J were obtained in this study. (Pseudopterogorgia = Antillogorgia). (DOCX) [file pone.0129609.s001.docx]

**S1 Figure**. **Phylogenetic tree of shallow-water octocorals in the Caribbean based on partial ND2 and mtMutS sequences.** The represented is the unrooted Bayesian Inference tree with its respective branch supports (estimated posterior probabilities). The bayesian inference, maximum credibility tree was the one with the highest branch support and best resolution in terminal branches although phylogenetic relationships among the three methods did not differ. Branches in boxes are zoomed aside for clarification purposes. Sequences that end with J were obtained in this study. (*Pseudopterogorgia* = *Antillogorgia*.). New sequences GenBank Accession Numbers KP777808-KP777816 (*ND2*) and KP772630- KP772634 (*mtMutS*). (Nexus tree below)


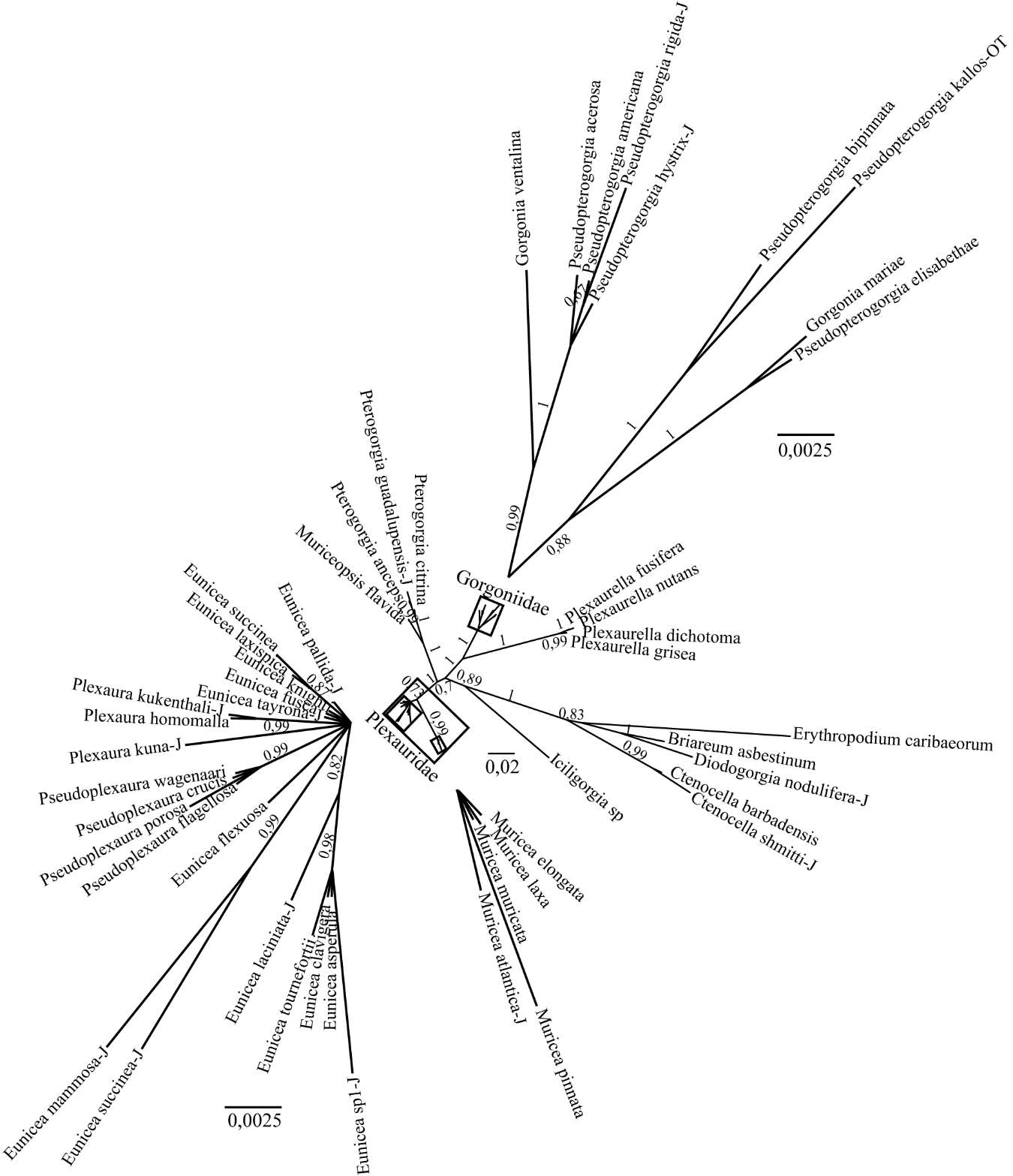


| #NEXUS  begin taxa;  dimensions ntax=49;  taxlabels  Pdichotom  Pgrisea  Pfusifera  Pnutans  Iciligosp  Ecaribaeo  Basbestin  DnodulifJ  Cbarbaden  CshmiJ  Melongata  Mlaxa  Mmuricata  Mpinnata  MatlanticJ  Esp1J  Easperula  Eclaviger  Etournefo  ElaciniaJ  EcalyculJ  EmammosaJ  Eflexuosa  Pflagello  Pporosa  Pcrucis  Pwagenaar  PkunaJ  Phomomall  PkukenthJ  EtayronaJ  Efusca  Eknighti  Elaxispic  Esuccinea  EpallidaJ  Mflavida  Panceps  PguadaJ  Pcitrina  Gventalin  Pacerosa  Pamerican  PrigidaJ  PhystrixJ  Pbipinnat  Pkallos  Gmariae  Pelisabet  ;  end;  begin trees;  tree con_50_majrule = [&R] (Pdichotom:0.001115,Pgrisea:0.001323,((Pfusifera:0.001291,Pnutans:0.004411)[&label=1.0]:0.006005,(((Iciligosp:0.072993,((Ecaribaeo:0.138279,(Basbestin:0.030032,DnodulifJ:0.050609)[&label=1.0]:0.026045)[&label=0.83]:0.009691,(Cbarbaden:0.006518,CshmiJ:0.029546)[&label=0.99]:0.064878)[&label=1.0]:0.071335)[&label=0.89]:0.013918,(((Melongata:0.001195,Mlaxa:0.001322,Mmuricata:0.001146,Mpinnata:0.007364,MatlanticJ:0.003328)[&label=0.99]:0.029658,(((Esp1J:0.008089,Easperula:0.001059,Eclaviger:0.001147,Etournefo:0.002692)[&label=0.98]:0.003118,ElaciniaJ:0.004631)[&label=0.82]:0.002762,(EcalyculJ:0.00844,EmammosaJ:0.009096)[&label=0.99]:0.006892,Eflexuosa:0.004534,(Pflagello:0.001143,Pporosa:0.003184,Pcrucis:0.001193,Pwagenaar:0.001057)[&label=0.99]:0.003948,PkunaJ:0.006597,(Phomomall:0.001102,PkukenthJ:0.001144)[&label=0.99]:0.00373,EtayronaJ:0.001107,Efusca:0.001067,Eknighti:0.001062,(Elaxispic:0.001097,Esuccinea:0.002167)[&label=0.82]:0.001868,EpallidaJ:0.001075)[&label=0.73]:0.004385)[&label=1.0]:0.017869,(Mflavida:0.018766,((Panceps:0.005026,PguadaJ:0.011182)[&label=0.99]:0.006615,Pcitrina:0.005256)[&label=1.0]:0.017914)[&label=1.0]:0.02686)[&label=0.7]:0.005762)[&label=1.0]:0.017157,((Gventalin:0.007927,(Pacerosa:0.00292,(Pamerican:0.001135,PrigidaJ:0.005231)[&label=0.67]:0.001811,PhystrixJ:0.001871)[&label=1.0]:0.005011)[&label=0.99]:0.00444,((Pbipinnat:0.005276,Pkallos:0.010112)[&label=1.0]:0.007268,(Gmariae:0.003209,Pelisabet:0.002151)[&label=1.0]:0.008757)[&label=0.88]:0.003202)[&label=1.0]:0.022228)[&label=1.0]:0.065694)[&label=0.99]:0.003834);  tree con_50_majrule = [&R] (Pdichotom:0.001115,Pgrisea:0.001323,((Pfusifera:0.001291,Pnutans:0.004411):0.006005,(((Iciligosp:0.072993,((Ecaribaeo:0.138279,(Basbestin:0.030032,DnodulifJ:0.050609):0.026045):0.009691,(Cbarbaden:0.006518,CshmiJ:0.029546):0.064878):0.071335):0.013918,(((Melongata:0.001195,Mlaxa:0.001322,Mmuricata:0.001146,Mpinnata:0.007364,MatlanticJ:0.003328):0.029658,(((Esp1J:0.008089,Easperula:0.001059,Eclaviger:0.001147,Etournefo:0.002692):0.003118,ElaciniaJ:0.004631):0.002762,(EcalyculJ:0.00844,EmammosaJ:0.009096):0.006892,Eflexuosa:0.004534,(Pflagello:0.001143,Pporosa:0.003184,Pcrucis:0.001193,Pwagenaar:0.001057):0.003948,PkunaJ:0.006597,(Phomomall:0.001102,PkukenthJ:0.001144):0.00373,EtayronaJ:0.001107,Efusca:0.001067,Eknighti:0.001062,(Elaxispic:0.001097,Esuccinea:0.002167):0.001868,EpallidaJ:0.001075):0.004385):0.017869,(Mflavida:0.018766,((Panceps:0.005026,PguadaJ:0.011182):0.006615,Pcitrina:0.005256):0.017914):0.02686):0.005762):0.017157,((Gventalin:0.007927,(Pacerosa:0.00292,(Pamerican:0.001135,PrigidaJ:0.005231):0.001811,PhystrixJ:0.001871):0.005011):0.00444,((Pbipinnat:0.005276,Pkallos:0.010112):0.007268,(Gmariae:0.003209,Pelisabet:0.002151):0.008757):0.003202):0.022228):0.065694):0.003834);  end;  begin figtree;  set appearance.backgroundColorAttribute="User Selection";  set appearance.backgroundColour=#-1;  set appearance.branchColorAttribute="User Selection";  set appearance.branchLineWidth=1.0;  set appearance.foregroundColour=#-16777216;  set appearance.selectionColour=#-2144520576;  set branchLabels.colorAttribute="User Selection";  set branchLabels.displayAttribute="Branch times";  set branchLabels.fontName="sansserif";  set branchLabels.fontSize=8;  set branchLabels.fontStyle=0;  set branchLabels.isShown=true;  set branchLabels.significantDigits=2;  set layout.expansion=0;  set layout.layoutType="RECTILINEAR";  set layout.zoom=0;  set nodeBars.barWidth=4.0;  set nodeLabels.colorAttribute="User Selection";  set nodeLabels.displayAttribute="Node ages";  set nodeLabels.fontName="sansserif";  set nodeLabels.fontSize=8;  set nodeLabels.fontStyle=0;  set nodeLabels.isShown=false;  set nodeLabels.significantDigits=4;  set polarLayout.alignTipLabels=false;  set polarLayout.angularRange=0;  set polarLayout.rootAngle=0;  set polarLayout.rootLength=100;  set polarLayout.showRoot=true;  set radialLayout.spread=0.0;  set rectilinearLayout.alignTipLabels=false;  set rectilinearLayout.curvature=0;  set rectilinearLayout.rootLength=100;  set scale.offsetAge=0.0;  set scale.rootAge=1.0;  set scale.scaleFactor=1.0;  set scale.scaleRoot=false;  set scaleAxis.automaticScale=true;  set scaleAxis.fontSize=8.0;  set scaleAxis.isShown=false;  set scaleAxis.lineWidth=1.0;  set scaleAxis.majorTicks=1.0;  set scaleAxis.origin=0.0;  set scaleAxis.reverseAxis=false;  set scaleAxis.showGrid=true;  set scaleAxis.significantDigits=4;  set scaleBar.automaticScale=true;  set scaleBar.fontSize=10.0;  set scaleBar.isShown=true;  set scaleBar.lineWidth=1.0;  set scaleBar.scaleRange=0.0;  set scaleBar.significantDigits=4;  set tipLabels.colorAttribute="User Selection";  set tipLabels.displayAttribute="Names";  set tipLabels.fontName="sansserif";  set tipLabels.fontSize=8;  set tipLabels.fontStyle=0;  set tipLabels.isShown=true;  set tipLabels.significantDigits=4;  set trees.order=false;  set trees.orderType="increasing";  set trees.rooting=false;  set trees.rootingType="User Selection";  set trees.transform=false;  set trees.transformType="cladogram";  end; |
| --- |
