# Supplementary material for: Octocoral Species Assembly and Coexistence in Caribbean Coral Reefs
Source: PLoS One. 2015 Jul 15;10(7):e0129609. doi: 10.1371/journal.pone.0129609 (PMC4503594; doi:10.1371/journal.pone.0129609)
Supplement: S1 Table — Data standardized to obtain a 10 m2 species density for each habitat and reef (see methods for details). A. Abundances by reef. B. Abundances by habitat. (DOCX) [file pone.0129609.s002.docx]

**S1 Table.** **Octocoral community abundances**. Data standardized to obtain a 10 m^2^ species density for each habitat and reef. A. Abundances by reef. B. Abundances by habitat.

A. Abundances by reef

| Species | Prov Barrier | AlBol Atoll | Quit Atoll | SM Dwr | Burb Dwr | Mon Dwr | Ime2 Dwr | Ime1 Dwr | PtaVen_Fringe |
| --- | --- | --- | --- | --- | --- | --- | --- | --- | --- |
| *Briareum asbestinum* | 12.75 | 1.29 | 2.60 | 0.00 | 0.00 | 0.00 | 0.00 | 0.00 | 0.00 |
| *Briareum polyanthes* | 1.45 | 0.24 | 0.06 | 0.00 | 0.00 | 0.00 | 0.00 | 0.00 | 0.00 |
| *Ctenocella barbadensis* | 0.00 | 0.00 | 0.00 | 0.00 | 0.00 | 3.00 | 5.50 | 1.17 | 0.00 |
| *Ctenocella schmitti* | 0.00 | 0.00 | 0.00 | 0.00 | 45.00 | 6.50 | 1.00 | 2.54 | 0.00 |
| *Diodogorgia nodulifera* | 0.00 | 0.00 | 0.00 | 0.00 | 0.00 | 3.00 | 0.00 | 0.92 | 0.00 |
| *Erythropodium caribaeorum* | 0.00 | 0.00 | 0.00 | 0.50 | 0.00 | 1.00 | 2.50 | 0.00 | 0.00 |
| *Eunicea asperula* | 0.00 | 0.00 | 0.00 | 5.00 | 2.00 | 2.50 | 5.50 | 0.00 | 0.00 |
| *Eunicea calyculata* | 0.26 | 0.00 | 0.06 | 0.50 | 0.00 | 0.50 | 0.00 | 0.00 | 0.00 |
| *Eunicea clavigera* | 0.63 | 0.06 | 0.70 | 1.50 | 3.00 | 8.00 | 10.50 | 1.92 | 0.20 |
| *Eunicea flexuosa* | 12.07 | 0.59 | 1.13 | 28.00 | 6.00 | 41.50 | 21.50 | 2.13 | 23.20 |
| *Eunicea fusca* | 0.00 | 0.12 | 0.68 | 34.50 | 51.00 | 8.00 | 2.50 | 0.00 | 1.80 |
| *Eunicea knigthi* | 0.00 | 0.06 | 0.00 | 0.00 | 2.50 | 0.00 | 0.50 | 0.00 | 0.00 |
| *Eunicea laciniata* | 0.05 | 0.00 | 0.15 | 1.00 | 6.00 | 0.00 | 0.00 | 0.00 | 0.00 |
| *Eunicea laxispica* | 0.38 | 0.00 | 0.13 | 0.50 | 1.50 | 0.00 | 0.00 | 0.00 | 0.00 |
| *Eunicea mammosa* | 4.09 | 0.18 | 0.74 | 0.50 | 2.50 | 0.00 | 0.00 | 0.25 | 0.00 |
| *Eunicea pallida* | 0.00 | 0.00 | 0.09 | 0.50 | 0.00 | 0.00 | 0.00 | 0.00 | 0.00 |
| *Eunicea sp1* | 0.00 | 0.00 | 0.00 | 4.50 | 0.00 | 9.00 | 4.00 | 0.00 | 0.00 |
| *Eunicea succinea* | 2.07 | 0.35 | 0.57 | 4.00 | 3.00 | 0.00 | 1.00 | 0.00 | 0.00 |
| *Eunicea tayrona* | 0.71 | 0.00 | 1.34 | 2.50 | 1.00 | 0.00 | 0.50 | 0.00 | 0.60 |
| *Eunicea tourneforti* | 1.69 | 0.06 | 0.60 | 1.00 | 2.00 | 0.00 | 0.00 | 0.00 | 3.00 |
| *Gorgonia mariae* | 0.18 | 0.00 | 0.55 | 0.00 | 0.00 | 0.00 | 0.00 | 0.00 | 0.00 |
| *Gorgonia ventalina* | 5.18 | 0.00 | 1.00 | 0.00 | 0.00 | 0.00 | 0.00 | 0.00 | 2.00 |
| *Iciligorgia schrammi* | 0.00 | 0.00 | 0.00 | 0.00 | 0.50 | 0.00 | 0.00 | 0.00 | 0.00 |
| *Muricea atlantica* | 0.00 | 0.00 | 0.09 | 0.00 | 0.00 | 0.00 | 0.00 | 0.00 | 0.00 |
| *Muricea elongata* | 0.00 | 0.00 | 0.30 | 0.00 | 0.50 | 0.00 | 0.00 | 0.00 | 0.00 |
| *Muricea laxa* | 0.00 | 0.00 | 0.00 | 0.00 | 0.00 | 13.00 | 1.00 | 0.75 | 0.00 |
| *Muricea muricata* | 0.00 | 0.00 | 0.21 | 1.00 | 0.00 | 0.00 | 0.00 | 0.00 | 0.00 |
| *Muricea pinnata* | 0.00 | 0.00 | 0.02 | 5.50 | 0.00 | 1.00 | 0.50 | 0.00 | 0.00 |
| *Muriceopsis flavida* | 1.98 | 0.53 | 2.49 | 0.50 | 1.50 | 0.00 | 0.00 | 0.00 | 0.20 |
| *Plexaura homomalla* | 10.06 | 0.12 | 1.30 | 0.00 | 0.00 | 0.00 | 0.00 | 0.00 | 25.20 |
| *Plexaura kukenthali* | 0.00 | 0.00 | 1.77 | 0.50 | 2.00 | 0.00 | 1.00 | 0.21 | 3.60 |
| *Plexaura kuna* | 0.00 | 0.00 | 0.02 | 0.00 | 0.00 | 0.00 | 0.00 | 0.00 | 0.00 |
| *Plexaurella dichotoma* | 2.22 | 0.53 | 0.34 | 0.00 | 0.00 | 0.00 | 0.50 | 0.00 | 0.40 |
| *Plexaurella fusifera* | 0.23 | 0.00 | 0.34 | 0.00 | 0.00 | 0.00 | 0.00 | 0.00 | 0.00 |
| *Plexaurella grisea* | 0.96 | 0.00 | 2.17 | 0.50 | 0.50 | 0.00 | 0.00 | 0.00 | 0.40 |
| *Plexaurella nutans* | 0.26 | 0.00 | 0.17 | 1.50 | 0.00 | 1.00 | 0.00 | 0.00 | 0.00 |
| *Pseudoplexaura crucis* | 0.00 | 0.00 | 0.28 | 0.00 | 0.00 | 0.00 | 0.00 | 0.00 | 0.00 |
| *Pseudoplexaura flagellosa* | 4.24 | 0.12 | 0.00 | 0.00 | 1.00 | 0.00 | 0.00 | 0.00 | 0.00 |
| *Pseudoplexaura porosa* | 7.48 | 0.24 | 0.00 | 0.00 | 0.50 | 0.00 | 0.00 | 0.00 | 5.00 |
| *Pseudoplexaura wagenaari* | 4.99 | 0.24 | 0.00 | 1.00 | 0.00 | 0.00 | 0.00 | 0.00 | 5.00 |
| *Pseudopterogorgia acerosa* | 0.06 | 0.65 | 0.19 | 1.00 | 3.00 | 1.00 | 0.50 | 1.58 | 0.00 |
| *Pseudopterogorgia americana* | 4.69 | 0.18 | 2.57 | 1.50 | 2.00 | 2.50 | 0.00 | 0.00 | 0.40 |
| *Pseudopterogorgia bipinnata* | 4.47 | 7.24 | 9.21 | 0.00 | 0.00 | 0.00 | 0.00 | 0.00 | 0.00 |
| *Pseudopterogorgia elisabethae* | 0.30 | 0.06 | 0.00 | 0.00 | 0.00 | 0.00 | 0.00 | 0.00 | 0.40 |
| *Pseudopterogorgia hystrix* | 2.19 | 0.00 | 0.00 | 0.00 | 0.00 | 0.00 | 0.00 | 0.00 | 0.00 |
| *Pseudopterogorgia kallos* | 0.25 | 0.06 | 0.00 | 0.00 | 0.00 | 0.00 | 0.00 | 0.00 | 0.00 |
| *Pseudopterogorgia rigida* | 0.60 | 0.00 | 0.00 | 0.00 | 0.00 | 0.00 | 0.50 | 0.00 | 1.00 |
| *Pterogorgia anceps* | 0.74 | 0.12 | 0.04 | 10.00 | 5.00 | 0.00 | 0.00 | 0.00 | 0.00 |
| *Pterogorgia citrina* | 1.08 | 0.00 | 0.60 | 38.00 | 16.00 | 0.00 | 0.00 | 0.00 | 0.00 |
| *Pterogorgia guadalupensis* | 0.05 | 0.00 | 0.00 | 0.00 | 1.00 | 0.00 | 0.00 | 0.00 | 0.00 |

B. Abundances by habitat.

| Species | Prov_Lag | Prov_Fore | AlBol_Lag | AlBol_Fore | Quit_Lag | Quit_Fore | Ronc_Lag | Ser_Lag | Burb_Plat | Burb_Slope | Mon_Plat | Mon_Slope | Ime2_Plat | Ime2_Slope | Ime1_Plat | Ime_Slope | SM_16mt | SM_5mt | PtaVen_Inner |
| --- | --- | --- | --- | --- | --- | --- | --- | --- | --- | --- | --- | --- | --- | --- | --- | --- | --- | --- | --- |
| *Briareum asbestinum* | 27.27 | 3.35 | 1.55 | 0.83 | 4.32 | 0.05 | 12.41 | 3.28 | 0.00 | 0.00 | 0.00 | 0.00 | 0.00 | 0.00 | 0.00 | 0.00 | 0.00 | 0.00 | 0.00 |
| *Ctenocella barbadensis* | 0.00 | 0.00 | 0.00 | 0.00 | 0.00 | 0.00 | 0.00 | 0.00 | 0.00 | 0.00 | 2.00 | 4.00 | 6.00 | 5.00 | 0.50 | 1.83 | 0.00 | 0.00 | 0.00 |
| *Ctenocella schmitti* | 0.00 | 0.00 | 0.00 | 0.00 | 0.00 | 0.00 | 0.00 | 0.00 | 0.00 | 90.00 | 0.00 | 13.00 | 0.00 | 2.00 | 0.50 | 4.58 | 0.00 | 0.00 | 0.00 |
| *Diodogorgia nodulifera* | 0.00 | 0.00 | 0.00 | 0.00 | 0.00 | 0.00 | 0.00 | 0.00 | 0.00 | 0.00 | 0.00 | 6.00 | 0.00 | 0.00 | 0.00 | 1.83 | 0.00 | 0.00 | 0.00 |
| *Erythropodium caribaeorum* | 0.00 | 0.00 | 0.00 | 0.00 | 0.00 | 0.00 | 0.00 | 0.00 | 0.00 | 0.00 | 2.00 | 0.00 | 4.00 | 1.00 | 0.00 | 0.00 | 1.00 | 0.00 | 0.00 |
| *Eunicea asperula* | 0.00 | 0.00 | 0.00 | 0.00 | 0.18 | 0.05 | 0.09 | 0.06 | 4.00 | 0.00 | 3.00 | 2.00 | 11.00 | 0.00 | 0.00 | 0.00 | 10.00 | 0.00 | 0.00 |
| *Eunicea calyculata* | 0.00 | 0.42 | 0.00 | 0.00 | 0.00 | 0.16 | 0.00 | 0.02 | 0.00 | 0.00 | 1.00 | 0.00 | 0.00 | 0.00 | 0.00 | 0.00 | 0.00 | 1.00 | 0.00 |
| *Eunicea clavigera* | 0.21 | 0.89 | 0.00 | 0.17 | 0.82 | 0.53 | 0.23 | 0.04 | 5.00 | 1.00 | 12.00 | 4.00 | 20.00 | 1.00 | 3.50 | 0.33 | 3.00 | 0.00 | 0.20 |
| *Eunicea flexuosa* | 10.05 | 13.38 | 0.64 | 0.00 | 1.18 | 1.05 | 0.27 | 0.43 | 10.00 | 2.00 | 41.00 | 42.00 | 42.00 | 1.00 | 3.00 | 1.25 | 55.00 | 1.00 | 23.20 |
| *Eunicea fusca* | 0.00 | 0.00 | 0.18 | 0.00 | 0.32 | 1.21 | 3.59 | 0.23 | 101.00 | 1.00 | 16.00 | 0.00 | 5.00 | 0.00 | 0.00 | 0.00 | 69.00 | 0.00 | 1.80 |
| *Eunicea knigthi* | 0.00 | 0.00 | 0.00 | 0.17 | 0.00 | 0.00 | 0.23 | 0.00 | 5.00 | 0.00 | 0.00 | 0.00 | 1.00 | 0.00 | 0.00 | 0.00 | 0.00 | 0.00 | 0.00 |
| *Eunicea laciniata* | 0.00 | 0.09 | 0.00 | 0.00 | 0.18 | 0.11 | 0.23 | 0.11 | 12.00 | 0.00 | 0.00 | 0.00 | 0.00 | 0.00 | 0.00 | 0.00 | 2.00 | 0.00 | 0.00 |
| *Eunicea laxispica* | 0.00 | 0.62 | 0.00 | 0.00 | 0.18 | 0.05 | 0.73 | 0.00 | 3.00 | 0.00 | 0.00 | 0.00 | 0.00 | 0.00 | 0.00 | 0.00 | 1.00 | 0.00 | 0.00 |
| *Eunicea mammosa* | 6.08 | 2.80 | 0.09 | 0.33 | 0.89 | 0.53 | 0.50 | 0.11 | 5.00 | 0.00 | 0.00 | 0.00 | 0.00 | 0.00 | 0.50 | 0.00 | 1.00 | 0.00 | 0.00 |
| *Eunicea pallida* | 0.00 | 0.00 | 0.00 | 0.00 | 0.11 | 0.05 | 0.05 | 0.02 | 0.00 | 0.00 | 0.00 | 0.00 | 0.00 | 0.00 | 0.00 | 0.00 | 1.00 | 0.00 | 0.00 |
| *Eunicea sp1* | 0.00 | 0.00 | 0.00 | 0.00 | 0.00 | 0.00 | 0.00 | 0.00 | 0.00 | 0.00 | 15.00 | 3.00 | 8.00 | 0.00 | 0.00 | 0.00 | 9.00 | 0.00 | 0.00 |
| *Eunicea succinea* | 1.95 | 2.14 | 0.45 | 0.17 | 2.00 | 0.37 | 0.05 | 0.15 | 5.00 | 1.00 | 0.00 | 0.00 | 2.00 | 0.00 | 0.00 | 0.00 | 8.00 | 0.00 | 0.00 |
| *Eunicea tayrona* | 0.71 | 0.72 | 0.00 | 0.00 | 0.29 | 1.05 | 0.23 | 0.04 | 2.00 | 0.00 | 0.00 | 0.00 | 1.00 | 0.00 | 0.00 | 0.00 | 4.00 | 1.00 | 0.60 |
| *Eunicea tourneforti* | 0.65 | 2.35 | 0.00 | 0.17 | 0.18 | 1.16 | 0.05 | 0.04 | 4.00 | 0.00 | 0.00 | 0.00 | 0.00 | 0.00 | 0.00 | 0.00 | 1.00 | 1.00 | 3.00 |
| *Gorgonia mariae* | 0.00 | 0.29 | 0.00 | 0.00 | 0.36 | 0.84 | 0.14 | 0.04 | 0.00 | 0.00 | 0.00 | 0.00 | 0.00 | 0.00 | 0.00 | 0.00 | 0.00 | 0.00 | 0.00 |
| *Gorgonia ventalina* | 2.02 | 7.22 | 0.00 | 0.00 | 0.61 | 1.58 | 1.64 | 0.98 | 0.00 | 0.00 | 0.00 | 0.00 | 0.00 | 0.00 | 0.00 | 0.00 | 0.00 | 0.00 | 2.00 |
| *Iciligorgia schrammi* | 0.00 | 0.00 | 0.00 | 0.00 | 0.00 | 0.00 | 0.00 | 0.00 | 0.00 | 1.00 | 0.00 | 0.00 | 0.00 | 0.00 | 0.00 | 0.00 | 0.00 | 0.00 | 0.00 |
| *Muricea atlantica* | 0.00 | 0.00 | 0.00 | 0.00 | 0.04 | 0.16 | 0.09 | 0.06 | 0.00 | 0.00 | 0.00 | 0.00 | 0.00 | 0.00 | 0.00 | 0.00 | 0.00 | 0.00 | 0.00 |
| *Muricea elongata* | 0.00 | 0.00 | 0.00 | 0.00 | 0.39 | 0.16 | 0.14 | 0.00 | 1.00 | 0.00 | 0.00 | 0.00 | 0.00 | 0.00 | 0.00 | 0.00 | 0.00 | 0.00 | 0.00 |
| *Muricea laxa* | 0.00 | 0.00 | 0.00 | 0.00 | 0.00 | 0.00 | 0.09 | 0.00 | 0.00 | 0.00 | 16.00 | 10.00 | 2.00 | 0.00 | 1.00 | 0.50 | 0.00 | 0.00 | 0.00 |
| *Muricea muricata* | 0.00 | 0.00 | 0.00 | 0.00 | 0.21 | 0.21 | 0.14 | 0.13 | 0.00 | 0.00 | 0.00 | 0.00 | 0.00 | 0.00 | 0.00 | 0.00 | 0.00 | 2.00 | 0.00 |
| *Muricea pinnata* | 0.00 | 0.00 | 0.00 | 0.00 | 0.04 | 0.00 | 0.00 | 0.00 | 0.00 | 0.00 | 1.00 | 1.00 | 1.00 | 0.00 | 0.00 | 0.00 | 11.00 | 0.00 | 0.00 |
| *Muriceopsis flavida* | 0.60 | 2.87 | 0.73 | 0.17 | 2.39 | 2.63 | 0.68 | 0.55 | 3.00 | 0.00 | 0.00 | 0.00 | 0.00 | 0.00 | 0.00 | 0.00 | 1.00 | 0.00 | 0.20 |
| *Plexaura homomalla* | 10.80 | 9.58 | 0.18 | 0.50 | 2.11 | 0.11 | 0.27 | 0.40 | 0.00 | 0.00 | 0.00 | 0.00 | 0.00 | 0.00 | 0.00 | 0.00 | 0.00 | 0.00 | 25.20 |
| *Plexaura kukenthali* | 0.00 | 0.00 | 0.00 | 0.00 | 2.07 | 1.32 | 0.91 | 0.62 | 4.00 | 0.00 | 0.00 | 0.00 | 2.00 | 0.00 | 0.00 | 0.42 | 1.00 | 0.00 | 3.60 |
| *Plexaura kuna* | 0.00 | 0.00 | 0.00 | 0.00 | 0.04 | 0.00 | 0.05 | 0.06 | 0.00 | 0.00 | 0.00 | 0.00 | 0.00 | 0.00 | 0.00 | 0.00 | 0.00 | 0.00 | 0.00 |
| *Plexaurella dichotoma* | 3.27 | 1.54 | 0.09 | 0.00 | 0.32 | 0.37 | 0.18 | 0.13 | 0.00 | 0.00 | 0.00 | 0.00 | 1.00 | 0.00 | 0.00 | 0.00 | 0.00 | 0.00 | 0.40 |
| *Plexaurella fusifera* | 0.00 | 0.37 | 0.00 | 0.00 | 0.11 | 0.68 | 0.14 | 0.06 | 0.00 | 0.00 | 0.00 | 0.00 | 0.00 | 0.00 | 0.00 | 0.00 | 0.00 | 0.00 | 0.00 |
| *Plexaurella grisea* | 0.83 | 1.05 | 0.00 | 0.00 | 3.29 | 0.53 | 1.73 | 0.64 | 1.00 | 0.00 | 0.00 | 0.00 | 0.00 | 0.00 | 0.00 | 0.00 | 0.00 | 1.00 | 0.40 |
| *Plexaurella nutans* | 0.00 | 0.44 | 0.00 | 0.00 | 0.14 | 0.21 | 0.32 | 0.19 | 0.00 | 0.00 | 2.00 | 0.00 | 0.00 | 0.00 | 0.00 | 0.00 | 3.00 | 0.00 | 0.00 |
| *Pseudoplexaura crucis* | 0.00 | 0.00 | 0.00 | 0.00 | 0.36 | 0.16 | 0.00 | 0.00 | 0.00 | 0.00 | 0.00 | 0.00 | 0.00 | 0.00 | 0.00 | 0.00 | 0.00 | 0.00 | 0.00 |
| *Pseudoplexaura flagellosa* | 4.22 | 4.26 | 0.09 | 0.17 | 0.00 | 0.00 | 0.00 | 0.00 | 2.00 | 0.00 | 0.00 | 0.00 | 0.00 | 0.00 | 0.00 | 0.00 | 0.00 | 0.00 | 0.00 |
| *Pseudoplexaura porosa* | 4.66 | 9.31 | 0.18 | 0.33 | 0.00 | 0.00 | 0.00 | 0.00 | 1.00 | 0.00 | 0.00 | 0.00 | 0.00 | 0.00 | 0.00 | 0.00 | 0.00 | 0.00 | 5.00 |
| *Pseudoplexaura wagenaari* | 3.26 | 6.11 | 0.36 | 0.00 | 0.00 | 0.00 | 0.00 | 0.00 | 0.00 | 0.00 | 0.00 | 0.00 | 0.00 | 0.00 | 0.00 | 0.00 | 2.00 | 0.00 | 5.00 |
| *Pseudopterogorgia acerosa* | 0.00 | 0.11 | 0.00 | 1.83 | 0.00 | 0.47 | 0.00 | 0.00 | 6.00 | 0.00 | 1.00 | 1.00 | 1.00 | 0.00 | 1.00 | 2.17 | 0.00 | 2.00 | 0.00 |
| *Pseudopterogorgia americana* | 3.36 | 5.54 | 0.09 | 0.33 | 0.86 | 5.11 | 3.95 | 1.85 | 4.00 | 0.00 | 5.00 | 0.00 | 0.00 | 0.00 | 0.00 | 0.00 | 1.00 | 2.00 | 0.40 |
| *Pseudopterogorgia bipinnata* | 6.51 | 3.14 | 7.27 | 7.17 | 7.36 | 11.95 | 63.36 | 10.0 | 0.00 | 0.00 | 0.00 | 0.00 | 0.00 | 0.00 | 0.00 | 0.00 | 0.00 | 0.00 | 0.00 |
| *Pseudopterogorgia elisabethae* | 0.00 | 1.50 | 0.00 | 0.17 | 0.00 | 0.00 | 2.64 | 0.89 | 0.00 | 0.00 | 0.00 | 0.00 | 0.00 | 0.00 | 0.00 | 0.00 | 0.00 | 0.00 | 0.40 |
| *Pseudopterogorgia hystrix* | 0.00 | 0.50 | 0.00 | 0.00 | 0.00 | 0.00 | 0.00 | 0.00 | 0.00 | 0.00 | 0.00 | 0.00 | 0.00 | 0.00 | 0.00 | 0.00 | 0.00 | 0.00 | 0.00 |
| *Pseudopterogorgia kallos* | 0.22 | 3.46 | 0.00 | 0.17 | 0.00 | 0.00 | 0.00 | 0.00 | 0.00 | 0.00 | 0.00 | 0.00 | 0.00 | 0.00 | 0.00 | 0.00 | 0.00 | 0.00 | 0.00 |
| *Pseudopterogorgia rigida* | 0.00 | 0.41 | 0.00 | 0.00 | 0.00 | 0.00 | 0.00 | 0.00 | 0.00 | 0.00 | 0.00 | 0.00 | 1.00 | 0.00 | 0.00 | 0.00 | 0.00 | 0.00 | 1.00 |
| *Pterogorgia anceps* | 0.00 | 1.22 | 0.00 | 0.33 | 0.04 | 0.05 | 0.00 | 0.00 | 10.00 | 0.00 | 0.00 | 0.00 | 0.00 | 0.00 | 0.00 | 0.00 | 0.00 | 20.00 | 0.00 |
| *Pterogorgia citrina* | 0.00 | 1.77 | 0.00 | 0.00 | 0.07 | 1.37 | 0.00 | 0.00 | 32.00 | 0.00 | 0.00 | 0.00 | 0.00 | 0.00 | 0.00 | 0.00 | 1.00 | 75.00 | 0.00 |
| *Pterogorgia guadalupensis* | 0.00 | 0.09 | 0.00 | 0.00 | 0.00 | 0.00 | 0.00 | 0.00 | 2.00 | 0.00 | 0.00 | 0.00 | 0.00 | 0.00 | 0.00 | 0.00 | 0.00 | 0.00 | 0.00 |
