# Supplementary material for: Octocoral Species Assembly and Coexistence in Caribbean Coral Reefs
Source: PLoS One. 2015 Jul 15;10(7):e0129609. doi: 10.1371/journal.pone.0129609 (PMC4503594; doi:10.1371/journal.pone.0129609)
Supplement: S2 Table — Colony shape, Branch type, Branching plane and Zooxanthellae presence are discrete qualitative characters. Branch thickness (cm), Calice length (cm), Intercalice distance (cm) and Calice aperture (cm) are quantitative mean values. (DOCX) [file pone.0129609.s003.docx]

**S2 Table**. **Functional characters data.** Colony shape, Branch type, Branching plane and Zooxanthellae presence are discrete qualitative characters. Branch thickness (cm), Calice length (cm), Intercalice distance (cm) and Calice aperture (cm) are quantitative mean values.

| **Species** | **Colony shape** | **Colony shape** | **Branch type** | **Branch type** | **Branching plane** | **Branching plane** | **Zoox** | **Branch thickness (cm)** | **Calice length (cm)** | **Intercalice distance (cm)** | **Calice aperture (cm)** |
| --- | --- | --- | --- | --- | --- | --- | --- | --- | --- | --- | --- |
| *Briareum asbestinum* | encrusting | 1 | cylindrical | 1 | no branching | 1 | 1 | 0.95 | 0.061 | 0.121 | 0.062 |
| *Ctenocella barbadensis* | flagelliform | 2 | cylindrical | 1 | no branching | 1 | 0 | 0.341 | 0.107 | 0.051 | 0.056 |
| *Ctenocella schmitti* | pinnate | 3 | flattened | 2 | single | 2 | 0 | 0.148 | 0.068 | 0.044 | 0.049 |
| *Diodogorgia nodulifera* | pinnate | 3 | cylindrical | 1 | no branching | 1 | 0 | 0.208 | 0.075 | 0.113 | 0.071 |
| *Erythropodium caribaeorum* | encrusting | 1 | N/A |  | no branching | 1 | 1 | 1.640 | 0.086 | 0.220 | 0.116 |
| *Eunicea asperula* | candelabrum-like | 4 | cylindrical | 1 | multiple | 3 | 1 | 0.428 | 0.184 | 0.154 | 0.147 |
| *Eunicea calyculata* | bushy | 5 | cylindrical | 1 | multiple | 3 | 1 | 0.882 | 0.142 | 0.098 | 0.100 |
| *Eunicea clavigera* | bushy | 5 | cylindrical | 1 | multiple | 3 | 1 | 0.412 | 0.279 | 0.164 | 0.114 |
| *Eunicea flexuosa* | bushy | 5 | cylindrical | 1 | single | 2 | 1 | 0.395 | 0.053 | 0.048 | 0.037 |
| *Eunicea fusca* | bushy | 5 | cylindrical | 1 | multiple | 3 | 1 | 0.312 | 0.060 | 0.051 | 0.042 |
| *Eunicea knigthi* | bushy | 5 | cylindrical | 1 | multiple | 3 | 1 | 0.360 | 0.059 | 0.064 | 0.082 |
| *Eunicea laciniata* | bushy | 5 | cylindrical | 1 | single | 2 | 1 | 1.187 | 0.182 | 0.067 | 0.145 |
| *Eunicea laxispica* | candelabrum-like | 4 | cylindrical | 1 | single | 2 | 1 | 0.266 | 0.232 | 0.154 | 0.123 |
| *Eunicea mammosa* | candelabrum-like | 4 | cylindrical | 1 | single | 2 | 1 | 0.445 | 0.167 | 0.054 | 0.081 |
| *Eunicea pallida* | bushy | 5 | cylindrical | 1 | multiple | 3 | 1 | 0.418 | 0.046 | 0.035 | 0.044 |
| *Eunicea sp1* | candelabrum-like | 4 | cylindrical | 1 | single | 2 | 1 | 0.325 | 0.129 | 0.088 | 0.105 |
| *Eunicea succinea* | candelabrum-like | 4 | cylindrical | 1 | single | 2 | 1 | 0.336 | 0.135 | 0.080 | 0.082 |
| *Eunicea tayrona* | bushy | 5 | cylindrical | 1 | single | 2 | 1 | 0.316 | 0.092 | 0.047 | 0.069 |
| *Eunicea tourneforti* | candelabrum-like | 4 | cylindrical | 1 | single | 2 | 1 | 0.915 | 0.255 | 0.065 | 0.119 |
| *Gorgonia mariae* | fan-like | 6 | flattened | 2 | single | 2 | 1 | 0.114 | 0.017 | 0.050 | 0.028 |
| *Gorgonia ventalina* | fan-like | 6 | flattened | 2 | single | 2 | 1 | 0.111 | 0.010 | 0.036 | 0.020 |
| *Iciligorgia schrammi* | fan-like | 6 | flattened | 2 | single | 2 | 0 | 0.194 | 0.074 | 0.082 | 0.075 |
| *Muricea atlantica* | candelabrum-like | 4 | cylindrical | 1 | single | 2 | 1 | 0.561 | 0.126 | 0.053 | 0.046 |
| *Muricea elongata* | bushy | 5 | cylindrical | 1 | multiple | 3 | 1 | 0.604 | 0.155 | 0.051 | 0.072 |
| *Muricea laxa* | bushy | 5 | cylindrical | 1 | multiple | 3 | 1 | 0.145 | 0.082 | 0.037 | 0.036 |
| *Muricea muricata* | candelabrum-like | 4 | cylindrical | 1 | single | 2 | 1 | 0.404 | 0.145 | 0.042 | 0.064 |
| *Muricea pinnata* | pinnate | 3 | cylindrical | 1 | single | 2 | 1 | 0.220 | 0.130 | 0.032 | 0.048 |
| *Muriceopsis flavida* | pinnate | 3 | cylindrical | 1 | multiple | 3 | 1 | 0.159 | 0.018 | 0.033 | 0.020 |
| *Plexaura homomalla* | bushy | 5 | cylindrical | 1 | multiple | 3 | 1 | 0.406 | 0.020 | 0.044 | 0.067 |
| *Plexaura kukenthali* | bushy | 5 | cylindrical | 1 | multiple | 3 | 1 | 0.285 | 0.020 | 0.052 | 0.077 |
| *Plexaura kuna* | bushy | 5 | cylindrical | 1 | multiple | 3 | 1 | 0.300 | 0.019 | 0.044 | 0.051 |
| *Plexaurella dichotoma* | bushy | 5 | cylindrical | 1 | multiple | 3 | 1 | 0.773 | 0.028 | 0.102 | 0.088 |
| *Plexaurella fusifera* | bushy | 5 | cylindrical | 1 | multiple | 3 | 1 | 1.008 | 0.033 | 0.111 | 0.078 |
| *Plexaurella grisea* | bushy | 5 | cylindrical | 1 | multiple | 3 | 1 | 0.546 | 0.026 | 0.057 | 0.089 |
| *Plexaurella nutans* | bushy | 5 | cylindrical | 1 | multiple | 3 | 1 | 1.430 | 0.069 | 0.108 | 0.161 |
| *Pseudoplexaura crucis* | bushy | 5 | cylindrical | 1 | multiple | 3 | 1 | N/A | N/A | N/A | N/A |
| *Pseudoplexaura flagellosa* | bushy | 5 | cylindrical | 1 | multiple | 3 | 1 | 0.377 | 0.028 | 0.050 | 0.089 |
| *Pseudoplexaura porosa* | bushy | 5 | cylindrical | 1 | multiple | 3 | 1 | 0.301 | 0.02 | 0.055 | 0.06 |
| *Pseudoplexaura wagenaari* | bushy | 5 | cylindrical | 1 | multiple | 3 | 1 | 0.379 | 0.032 | 0.048 | 0.105 |
| *Pseudopterogorgia acerosa* | feather-like | 7 | flattened | 2 | multiple | 3 | 1 | 0.183 | 0.022 | 0.052 | 0.078 |
| *Pseudopterogorgia americana* | feather-like | 7 | flattened | 2 | multiple | 3 | 1 | 0.128 | 0.025 | 0.023 | 0.045 |
| *Pseudopterogorgia bipinnata* | pinnate branched | 3 | flattened | 2 | single | 2 | 1 | 0.211 | 0.036 | 0.044 | 0.045 |
| *Pseudopterogorgia elisabethae* | pinnate branched | 3 | flattened | 2 | single | 2 | 1 | 0.093 | 0.022 | 0.062 | 0.053 |
| *Pseudopterogorgia hystrix* | pinnate branched | 3 | flattened | 2 | multiple | 3 | 1 | 0.124 | 0.014 | 0.034 | 0.064 |
| *Pseudopterogorgia kallos* | feather-like | 7 | flattened | 2 | single | 2 | 1 | 0.111 | 0.017 | 0.024 | 0.057 |
| *Pseudopterogorgia rigida* | feather-like | 7 | flattened | 2 | multiple | 3 | 1 | 0.146 | 0.030 | 0.025 | 0.058 |
| *Pterogorgia anceps* | bushy | 5 | flattened | 2 | multiple | 3 | 1 | 0.469 | 0.020 | 0.040 | 0.088 |
| *Pterogorgia citrina* | bushy | 5 | flattened | 2 | multiple | 3 | 1 | 0.229 | 0.029 | 0.041 | 0.104 |
| *Pterogorgia guadalupensis* | bushy | 5 | flattened | 2 | multiple | 3 | 1 | 0.541 | 0.024 | 0.097 | 0.101 |
