# Supplementary material for: Octocoral Species Assembly and Coexistence in Caribbean Coral Reefs
Source: PLoS One. 2015 Jul 15;10(7):e0129609. doi: 10.1371/journal.pone.0129609 (PMC4503594; doi:10.1371/journal.pone.0129609)
Supplement: S3 Table — (DOCX) [file pone.0129609.s004.docx]

**S3 Table**. Eight evaluated traits with their respective categories, K statistic and interpretation of their evolution.

| **Trait** | **Category** | **K statistic** | **Evolution** |
| --- | --- | --- | --- |
| Zooxanthellae | Presence/Absence | 3.9634 | Conserved |
| Branch shape | No branching | 1.5398 | Conserved |
|  | Cylindrical  Flattened |  |  |
| Branch plane | No branching | 0.2588 | Labile |
|  | Single |  |  |
|  | Multiple |  |  |
| Colony shape | Encrusting | 0.2266 | Labile |
|  | Flagelliform |  |  |
|  | Pinnate |  |  |
|  | Candelabrum-like |  |  |
|  | Bushy |  |  |
|  | Fan-like |  |  |
|  | Feather-like |  |  |
| Branch thickness | Continuous | 0.1631 | Labile |
| Intercalyce distance | Continuous | 0.1088 | Labile |
| Calyx length | Continuous | 0.0960 | Labile |
| Calyx aperture | Continuous | 0.0730 | Labile |
